# Geographical Factors Affecting Bed Net Ownership, a Tool for the Elimination of *Anopheles*-Transmitted Lymphatic Filariasis in Hard-to-Reach Communities

**DOI:** 10.1371/journal.pone.0053755

**Published:** 2013-01-07

**Authors:** Michelle C. Stanton, Moses J. Bockarie, Louise A. Kelly-Hope

**Affiliations:** Centre for Neglected Tropical Diseases, Liverpool School of Tropical Medicine, Liverpool, United Kingdom; Tulane University School of Public Health and Tropical Medicine, United States of America

## Abstract

Vector control, including the use of bed nets, is recommended as a possible strategy for eliminating lymphatic filariasis (LF) in post-conflict countries such as the Democratic Republic of Congo (DRC). This study examined the geographical factors that influence bed net ownership in DRC in order to identify hard-to-reach communities that need to be better targeted. In particular, urban/rural differences and the influence of population density, proximity to cities and health facilities, plus access to major transport networks were investigated. Demographic and Health Survey geo-referenced cluster level data were used to map bed net coverage (proportion of households with at least one of any type of bed net or at least one insecticide-treated net (ITN)), and ITN density (ITNs per person) for 260 clusters. Bivariate and multiple logistic or Poisson regression analyses were used to determine significant relationships. Overall, bed net (30%) and ITN (9%) coverage were very low with significant differences found between urban and rural clusters. In rural clusters, ITN coverage/density was positively correlated with population density (r = 0.25, 0.27 respectively, p<0.01), and negatively with the distance to the two largest cities, Kinshasa or Lubumbashi (r = −0.28, −0.30 respectively, p<0.0001). Further, ownership was significantly negatively correlated with distance to primary national roads and railways (all three measures), distance to main rivers (any bed net only) and distance to the nearest health facility (ITNs only). Logistic and Poisson regression models fitted to the rural cluster data indicated that, after controlling for measured covariates, ownership levels in the Bas-Congo province close to Kinshasa were much larger than that of other provinces. This was most noticeable when considering ITN coverage (odds ratio: 5.3, 95% CI: 3.67–7.70). This analysis provides key insights into the barriers of bed net ownership, which will help inform both LF and malaria bed net distribution campaigns as part of an integrated vector management strategy.

## Introduction

The goal of the Global Programme to Eliminate Lymphatic Filariasis (GPELF), established in 2000, is to eliminate lymphatic filariasis (LF) as a public health problem by 2020 [1]. This will primarily be achieved through the use of mass drug administration (MDA) of albendazole plus either ivermectin or diethylcarbamazine (DEC) to the at-risk population. Whilst the programme is showing signs of success in many LF endemic countries [1], more than half of the 32 LF countries in sub-Saharan Africa are yet to implement MDA, 11 years after the GPELF was launched. The majority of these countries are post-conflict countries with fragile health systems in a resource poor setting, recovering from the ravages of war. However, the main vector of LF in these hard-to-reach areas, the *Anopheles* mosquito, is very susceptible to insecticide-treated bed nets (ITNs) and long-lasting insecticide treated bed nets (LLINs). Efficient management of the distribution of bed nets could accelerate the elimination of LF in these areas despite the late start of MDA. Of these post-conflict countries, the Democratic Republic of Congo (DRC) has the heaviest burden of LF, with an at-risk population of almost 50 million [1].

Administrating MDA in a country such as DRC will be challenging due to the lack of human and financial resources, poor internal transport infrastructure, and the difficulties of remote inaccessible terrain, which is further complicated by *Loa loa* co-endemicity and the potential for severe adverse events (SAEs) in individuals with high microfilaria loads [2]–[4]. As a result, vector control is increasingly being considered as an alternative strategy for LF elimination, especially in sub-Saharan Africa where the vector is the *Anopheles* mosquito, and LF and malaria are co-endemic. Sufficiently high coverage of bed nets should reduce the transmission of both diseases [5]–[9], hence the World Health Organization (WHO) is advocating for integrated vector management (IVM) in these areas [10], [11]. A recent review has highlighted successful examples of IVM [12], and by jointly managing the vector control activities of both LF and malaria programmes, it should be possible to better target the delivery and coverage of bed nets, specifically ITNs, in hard-to-reach areas [13].

Bed net targets set by international malaria programmes include 80% coverage of ITNs for all age groups at risk of malaria [14], and one ITN per two people [15] amongst all age groups, often termed as ‘universal coverage’. It is well recognised that bed net distribution programmes are more successful at reaching urban areas in comparison to rural areas [16]–[20], and differences in coverage in DRC have previously been reported [4], [21], [22]. These differences may partly be attributed to urban communities being closer to transport networks and bed net distribution points [17], [18], [23], however the variability in ownership in urban and rural communities has not been explored in any detail. Several studies in sub-Saharan Africa found that proximity to health centres was a significant risk factor [19], [24], [25], and the small scale study in Kenya also found that households closer to roads (<3 km) were more likely to own a bed net than those further away [19]. The influence of these and other geographical factors has generally not been considered or examined collectively on a large scale.

Understanding barriers to the distribution of bed nets for LF elimination is critical in countries such as DRC, especially given its vast geographical size and potentially large overlapping *Loa loa* endemic areas [1], [4]. To begin to address these issues, this paper aimed to explore the influence of geographical factors such as human demography, infrastructure and transport networks on bed net ownership in DRC. Specifically, we examined the differences within and between urban and rural communities with respect to population density, distance to large cities, distance to health facilities and proximity to transport networks, including road, rail, river and air.

## Methods

### Data Sources

#### Bed net data

Demographic and Health surveys (DHS) [26] are designed to provide data for a wide range of monitoring and impact evaluation indicators in the areas of population, health and nutrition. Several surveys are undertaken within over any given survey period, at both the individual level, and the household level. The first DHS survey for DRC was conducted in 2007, and included questions on whether or not the household owned any bed nets, further to the number and type of bed net they owned. For the purposes of this study data were aggregated to the cluster (community) level, and for each cluster it was possible to derive three measures of i) any bed net coverage, defined as the proportion of surveyed households within a cluster that owned at least one of any type of bed net, ii) ITN coverage, defined as the proportion of surveyed households within a cluster that owned at least one ITN and iii) ITN density, defined as the number of ITNs per person amongst surveyed households within a cluster. A cluster level analysis, as opposed to a household level analysis, was undertaken due to the primary focus of this analysis being on the influence of geographical factors on access to bed nets across a large area, namely DRC. As such the small scale influences within a cluster were considered to be negligible.

In DRC, the DHS survey included a total of 300 community clusters. These clusters were selected using a stratified two-stage cluster design for statutory cities, and a stratified three-stage cluster design for all other areas, with the 1984 census and subsequent updates made to these figures being used as a sampling frame. Neighbourhoods were used as sampling units within statutory cities, whereas villages were used as sampling units outside of these areas. If the neighbourhood/village exceeded 500 household, the area was divided further, and only one of the divisions was used as the sampling unit [27]. Within the clusters, between 25 and 32 households were surveyed (median 30, total number of 8886 households) with the total number of people residing in each household between 1 and 28 (median 5). The GPS coordinates were recorded and verified for 293 of the 300 clusters. Those with missing GPS coordinates were excluded from the analysis. To ensure confidentiality, it is DHS policy to randomly displace the GPS coordinates for all surveyed clusters, with rural clusters displaced by 0 to 5 km (with 1% displaced by 0 to 10 km), and urban clusters displaced by 0 to 2 km. However, as our analysis considered distances on a large national scale, these artefacts data should not have an influence on the subsequent results especially given the large geographical size of DRC.

Clusters at an altitude of greater than 1500 km (n = 33) were excluded from the analysis resulting in 260 remaining. These high altitude clusters were in the eastern highlands within the Kivu and Katanga provinces where current and historical data indicate there is little evidence of LF transmission [4]. Furthermore, of the approximately 70 million people living in DRC, only 3% live in these mountainous areas whereas the remaining 97% live in equatorial forest and tropical zones and experience stable malaria transmission [28].

#### Urban/Rural classification

Despite the importance of urbanisation with respect to health outcomes, to date there is no common definition as to what constitutes an urban area, and subsequently what constitutes a rural area [20]. Therefore, we used two broad types of urban/rural classification, namely those that are locally defined, and those that are globally defined and as such are internationally comparable. The classifications used within the DHS data are generally defined by the national census data and as a result, cannot be readily compared between countries. In DRC, the most recent national survey was undertaken in 1984 so some urban/rural classifications are likely to be outdated. To account for this, we also examined globally defined urban/rural classification data obtained from the Global Rural Urban Mapping Project, referred to as GRUMP [29], [30]. The GRUMP urban extent map is a 1 km ×1 km map based on the combination of the most recent national census data and satellite that has been used to delineate urban areas such as the National Oceanic and Atmospheric Administration's (NOAA) night-time lights data set [31]. The urban extent map for DRC was obtained for the year 2000.

#### Human demography and infrastructure

To broadly examine the influence of human demography and infrastructure in relation to bed net ownership, the population density of each cluster, the distance to the major cities (Kinshasa, Lubumbashi, Kisangani), and the distance to the nearest health facility were examined. Population density estimates for each cluster were obtained at a spatial resolution of approximately 5 km ×5 km for 2005 from the Gridded Population of the World, version 3 (GPWv3, [32]). These estimates are based primarily on national census data and additional data obtained prior to 2000. GPWv3 projections for 2005 were produced in collaboration with the United Nations Food and Agriculture Organization (FAO). Further, the size (population estimates), and longitude and latitude of the towns/cities of DRC were obtained from the World Gazetteer [33], and was used to identify the town/city to which each of the urban clusters belonged. The Euclidean distances between each cluster and the nearest major city were measured in kilometres (km) using the proximity analysis tool in ArcGIS 10. The distance to health facility values were based on the DHS household data, which reported the amount of time (in minutes) it took a household to travel to the nearest health facility. The median reported time for each cluster was used to represent the cluster-level distance.

#### Transport networks

Transport network data, including national roads, railways, navigable waterways and airports were obtained from the United Nations Joint Logistic Centre (UNJLC) [34], the World Food Programme (WFP) Logistics Cluster and the United Nations Development Programme (UNDP) collated over the period 2006–2009. As the internal conflict and political unrest and associated economic collapse in DRC is likely to have had an adverse effect on the transport infrastructure, it was decided that only primary transport networks would be included in the analysis. The road network in DRC is comprised of 9 national roads in addition to priority regional roads, secondary regional roads and local roads. Only the main national roads (1 and 2) were considered as many others were classed as very degraded. Similarly, railways that were classed as degraded by the UNJLC in 2005–6 were excluded from the analysis, and only the primary navigable waterways were considered as opposed to all navigable waterways. The location of airports in DRC was also available, however it was not possible to differentiate between small and large airports, and those in regular use, so all airport were included in the analysis.

### Statistical and Spatial Analysis

All mapping, spatial and statistical analysis was undertaken in R (version 2.14.1) and/or ArcGIS 10 (ESRI Redlands, CA). The differences between urban and rural clusters were first compared, followed by separate analyses of urban and rural clusters. As per the DHS statistical guidelines [35], survey sampling weights have been taken into account when calculating bed net summary measures, but were not used in subsequent correlation and regression analyses.

#### Urban and Rural comparisons

The urban/rural classifications from the DHS and GRUMP data were first explored in order to determine how they differed, and to identify a dataset for which DHS and GRUMP urban/rural classifications agreed and could therefore be used in the subsequent analyses. Second, summaries of the cluster level minimum, lower quartile, median, upper quartile and maximum were produced for each of the three bed net measures i) bed net coverage, ii) ITN coverage and iii) ITN density, in addition to demographic and transport network variables by urban/rural classification. Survey weights were used to produce the summaries of bed net ownership measures. Due to the skewness of each measure, non-parametric Mann-Whitney U tests were used to determine whether the two groups differed.

#### Urban analysis

In order to examine bed net ownership in urban areas in more detail, first the median measures of i) bed net coverage, ii) ITN coverage and iii) ITN density in the three major cities namely, Kinshasa (pop. 7,273,947), Lubumbashi (pop. 1,283,380) and Kisangani (pop. 682,599) were summarised. Second, the influence of distance between each of the urban cluster and these three major cities was considered. The Spearman's rank correlation coefficient was calculated between the three bed net ownership measures and the distance to Kinshasa for urban clusters only. Combinations of cities (Kinshasa plus Lubumbashi, Kinshasa plus Kisangani, all three cities) were then considered e.g. if considering the distance from Kinshasa or Lubumbashi, the correlation was calculated between bed net ownership and the shortest of the two distances. In order to determine whether the relationship between bed net ownership and distance was linear, or whether the bigger influence was whether or not the cluster was within the cities themselves, the calculation was undertaken both including and excluding clusters that were within the cities. Further, correlation between the three bed net measures and the size of the town/city to which the urban cluster belonged was also calculated.

#### Rural analysis

In order to examine bed net ownership in rural areas and the influence of demographic and transports variables, the relationship between i) bed net coverage, ii) ITN coverage and iii) ITN density and the population density, and distance to Kinshasa, plus combinations of three major cities, to nearest health facility, and to nearest national road, railway, navigable river and airport networks were first determined by univariate analysis and calculating the Spearman's rank correlation coefficient.

Second, a logistic regression model was fitted to the coverage data to determine the combination of demographic and transport variables that best explain the variability in bed net coverage. Further, a Poisson regression model was fitted to the ITN density data such that the response variable was the number of ITNs per cluster, and the log-transformed population per cluster was included as an offset in the model. The variables included in the final models were selected using a stepwise backwards selection approach, with the full model containing the variables that were significantly correlated with the bed net measures in the univariate analysis. An assumption of both the logistic regression and Poisson regression modelling is that the models residuals (a measure of the deviation of the observed data from the fitted model) are independent. Any spatial autocorrelation in the model's residuals would indicate that the selected model does not fully explain the spatial variability in bed net ownership, and hence suggest that additional geographical variables may be needed. Therefore, the residuals of each of the regression model were tested for spatial autocorrelation using the global Moran's I statistic [36].

Further to explore the relationship between bed net ownership and each variable, interactions between the demographic and transport variables were considered, i.e. were more densely populated clusters close to cities more likely to own bed nets than less densely populated clusters close to the city? These interactions were explored firstly by categorising the clusters according to their geographical attributes and calculating the overall bed net ownership measures for each category. Then, more formally, interaction terms were added to the previously selected regression model, and the test for spatial autocorrelation in the residuals was repeated.

No ethical review committee approval has been obtained for this study as the analysis was based on published or otherwise publicly available information.

## Results

**Figure 1:**
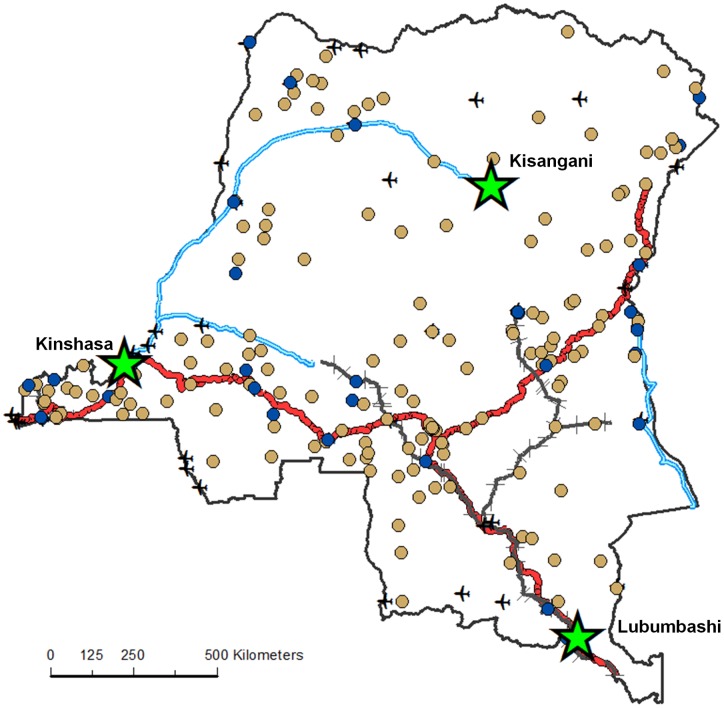
Locations of the DHS surveys with matching urban/rural definitions. Map of the DHS survey locations (urban = blue, rural = light brown) plus geographical features including cities with population greater than 500,000 (stars), national roads (red lines), navigable waterways (blue lines) and railways (black lines).

Figure 1 presents the location of the urban and rural clusters, the three largest cities and the main roads, rivers, railways and navigable waterways in DRC. This map demonstrates that the majority of the surveyed population are located in the more accessible south-eastern half of the country, with a small proportion of the population being sampled in the central region of the country where the Congo River basin dominates the landscape.

Overall, bed net coverage was very low at 30% (cluster median 27%, interquartile range (IQR) 10%–47%). Of the 260 clusters under consideration, 18% reported two or fewer households owning any bed nets. Further, overall ITN coverage was 9% (cluster median 7%, IQR 3%–14%) and the overall ITN density was 2 per 100 people (cluster median 1, IQR 0–3). Of the 260 clusters, 53% reported two or fewer households owning any ITNs.

### Urban and Rural comparisons

Table 1 presents summaries of each of the three bed net ownership measures and demographic and transport network variables stratified by urban and rural classes. In total, 223 of the 260 clusters with geographical information available agreed on DHS and GRUMP urban/rural classifications (Figure 1). Of those agreeing in classification, 79 were urban and 144 were rural. Summaries of the data stratified by the DHS urban/rural classification and GRUMP urban/rural classification are presented in the Table S1.

**Table 1:** Summaries of bed net measures, demographic features and transport networks by urban/rural classification.

	Urban (n = 79)					Rural (n = 144)				
Attribute	Min.	Q1	Med	Q3	Max	Min.	Q1	Med	Q3	Max
**Bed net summaries**										
** Bed net coverage**	3	20	43	54	87	0	7	17	40	100
** ITN coverage**	0	6	10	20	43	0	0	3	10	67
** ITN density (per 100 pop.)**	0	1	2	4	11	0	0	1	2	18
**Demographic features**										
** Population density (pop/km^2^)**	9	79	1112	7953	7953	2	10	23	41	901
** Distance to major city (km)**	1	7	18	580	841	51	308	496	629	798
** Distance to Kinshasa (km)**	1	7	14	412	1277	4	183	372	633	1292
** Distance to health facility (mins)**	5	10	15	20	60	3	15	55	120	510
**Transport networks**										
** Distance to main road (km)**	0	1	3	86	1002	0	39	86	227	998
** Distance to railway (km)**	0	7	16	87	983	1	56	146	242	918
** Distance to main waterway (km)**	0	7	13	361	678	1	131	253	379	763
** Distance to airport (km)**	1	4	7	11	187	4	76	114	155	270

Urban clusters had higher bed net ownership than rural clusters for all three bed net ownership measures. This difference was largest for bed net coverage, with a median proportion of 43% (IQR 20%–54%) observed for urban clusters compared with 17% (IQR 7%–40%) for rural clusters. For ITN-related summary measures, only small differences were observed between urban and rural clusters, due to the small number of households with ITNs throughout DRC. All differences were statistically significantly different with p-values less than 0.05.

Differences were also found with regards to demographic and transport features (Table 1). There were clear differences in the population density, and individuals in rural clusters were found to have to travel up to 500 km to reach one of the three major cities in comparison to 18 km in urban clusters (rural-median 496 km IQR 308–629; urban – median 18 km, IQR 7–580), and nearly four times as far to the nearest health facility (rural – median 55 mins, IQR 15–120; urban – median 15 mins, IQR 10–20) in contrast to those in urban clusters. Similarly, for transport networks, rural clusters were found to be between nine and twenty seven times further from the main national roads, railways, waterways and airports than rural clusters. Mann-Whitney U tests performed to compare all pairs of demographic and transport network variables were statistically significant (p<0.0001).

### Urban analysis

#### Differences within and between urban areas

A large amount of variability in bed net summary measures was observed among the urban clusters (Table 1). Bed net coverage ranged from 3% to 87%, ITN coverage ranged from 0% to 43% and ITN density per 100 people ranged from 0 to 11. Further, it was noted that bed net ownership data were negatively skewed for all three measures such that only a few clusters achieved high bed net ownership levels.

**Figure 2:**
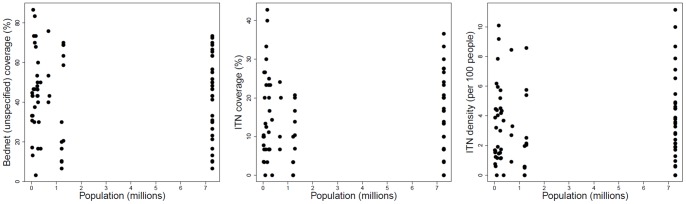
Bed net ownership of urban clusters by town/city size.

The differences between the bed net measures and the size of the town/city to which the urban cluster belonged indicated that 29 towns/cities were closest to the 79 urban clusters, with estimated population sizes ranging from 10,998 (Gungu, Bandundu province) to 7,273,947 (Kinshasa). Just over half (40/79) of these urban clusters are within the three major cities, primarily Kinshasa. Figure 2 presents a scatterplot of town/city size against each of the three bed net measures, with no clear relationship being visible. Correlation coefficients, both including and excluding the clusters in Kinshasa were non-significant.

Differences in bed net ownership were observed between clusters within the three largest cities, defined as being within 20 km of the city's centroid; Kinshasa (n = 31, median = 45% 17%, 3.4 per 100 people for bed net coverage, ITN coverage and ITN density respectively), Lubumbashi (n = 6, median = 63%, 13%, 2.5 per 100 people and Kisangani (n = 3, median = 40%, 7%, 0.9 per 100 people). Distances between urban clusters and the closest of the three largest cities ranged from 0.5 km to 840 km with a median of 12 km.

Correlations between the three bed net measures and the distances to Kinshasa and the different city combinations was found to be strongest for the Kinshasa/Lubumbashi combination i.e. the distance to Kinshasa, or to Lubumbashi if closer (correlation = −0.20, p = 0.0737; correlation  = −0.23, p = 0.0400; correlation = −0.29, p = 0.0095 for bed net coverage, ITN coverage and ITN density respectively). However, on excluding clusters that were in the cities themselves (i.e. within 20 km of the city's centroid) it was observed that the relationship between bed net ownership and distance to the closest of the two cities became non-significant (correlation = 0.10, p = 0.5137; correlation = −0.04, p = 0.7980; correlation = −0.09, p = 0.5793 for bed net coverage, ITN coverage and ITN density respectively). As such, cluster classified as “urban” could effectively be stratified into “city” and “non-city” clusters. Weighted median bed net measures for urban city clusters i.e. those within 20 km of the centroid of either Kinshasa (n = 31) or Lubumbashi (n = 6) were 47%, 16% and 3 per 100 people respectively, whereas in urban non-city clusters the median bed net measures were 40%, 10% and 2 per 100 people. These differences were significant for ITN density only.

### Rural Analysis

#### Correlations and maps

Table 2 presents the correlation coefficients between the three bed net measures in rural clusters, and demographic and transport factors. The strongest correlation (significantly negative) was found to be the distance to Kinshasa/Lubumbashi (correlation  = −0.39, p = 0.0222; correlation = −0.43, p<0.0001; correlation = −0.42, p<0.0001 for bed net coverage, ITN coverage and ITN density respectively), where the distances of rural clusters to Kinshasa/Lubumbashi ranged from 50 km to 1700 km, with a median of 750 km. Significant negative correlations were also found with population density, distance to health facility and the distances to the main national roads and railways. Of these, the relationships tended to be stronger for measures relating to ITN ownership. The correlation between the distance to the navigable waterway was only significant for any bed net coverage, while the distance to the nearest airport did not appear to be significant at all.

**Table 2:** Correlation coefficient, plus associated p-values between bed net ownership in rural clusters and demographic and transport attributes.

	Net ownership						
	Any bed net coverage			ITN coverage		ITN density	
**Demographic Features**							
** Pop. density (pop/km^2^)**	0.15		(0.0754)	0.25	(0.0029)	0.27	(0.0012)
** Distance to health facility (mins)**	−0.11		(0.1600)	−0.19	(0.0130)	−0.18	(0.0222)
** Distance to nearest major city (km)**	−0.26		(0.0017)	−0.38	(<0.0001)	−0.39	(<0.0001)
** Distance to Kinshasa (km)**	−0.24		(0.0036)	−0.31	(0.0002)	−0.31	(0.0002)
** Distance to Kinshasa/Kisangani (km)**	−0.15		(0.0801)	−0.28	(0.0007)	−0.30	(0.0003)
** Distance to Kinshasa/Lubumbashi (km)**	−0.39		(<0.0001)	−0.43	(<0.0001)	−0.42	(<0.0001)
**Transport Features**							
** Distance to national road (km)**	−0.22		(0.0070)	−0.31	(0.0002)	−0.31	(0.0002)
** Distance to railway (km)**	−0.24		(0.0045)	−0.32	(0.0001)	−0.31	(0.0001)
** Distance to main waterways (km)**	−0.19		(0.0246)	−0.09	(0.2840)	−0.09	(0.2574)
** Distance to airport (km)**	0.07		(0.3883)	0.01	(0.8994)	−0.01	(0.9049)

**Figure 3:**
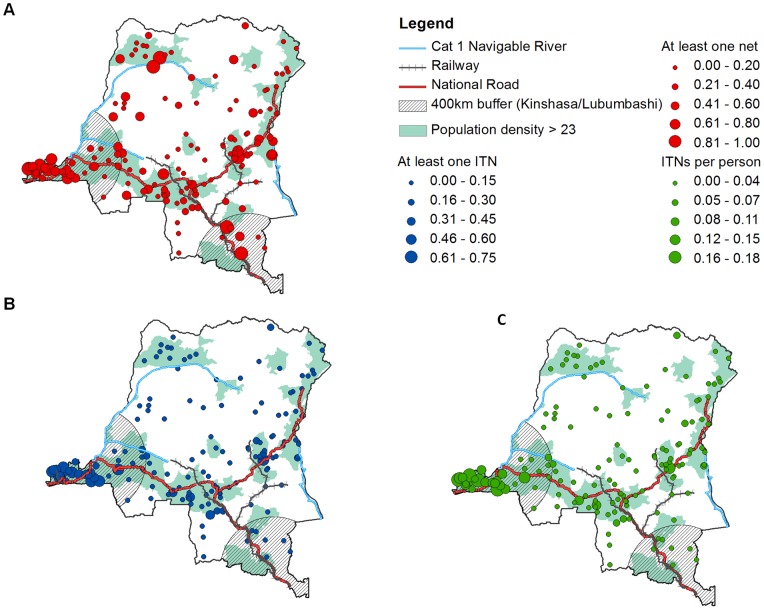
Maps of bed net coverage, ITN coverage and ITN density. Maps of rural DHS clusters, with the size of the circle representing A) any bed net coverage, B) ITN coverage and C) ITN density. The scored areas represent areas 400 km from Kinshasa and Lubumbashi and the blue regions represent areas with population density greater than the median 23.

Figure 3 presents maps of each of the three bed net ownership measures and distinguishes areas within 400 km to Kinshasa and Lubumbashi, and areas of a higher median population density >23. The main national roads, railways, and navigable waterways are also highlighted. In each of the three maps the higher measures in the Bas-Congo in the far western side of the country is clearly visible. For any bed net coverage, the trend for the remainder of the country is less clear, although values tend to be higher along the main national roads, and in areas in the Congo basin that are closest to the river. For both ITN measures, outside of the Bas-Congo and the area surrounding Kinshasa, ITN ownership is low, with higher levels of ITN ownership occurring sporadically in the more highly densely populated areas in the southern half of the country.

#### Regression models

The logistic regression models for any bed net coverage included the distance to Kinshasa/Lubumbashi and the main roads, railways and navigable waterways as explanatory variables, with the likelihood of ownership decreasing as the distance to a main road, railway and waterway increased. The selected models for ITN ownership measures included population density and distance to Kinshasa/Lubumbashi, the nearest health facility, and railway as explanatory variables, with the likelihood of owning an ITN increasing as population density increased, and decreased as the distance to Kinshasa/Lubumbashi, the nearest health facility or railway increased. All explanatory variables were log transformed in order to reduce the amount of skew that was observed, and therefore improve the fit of the resulting model.

**Figure 4:**
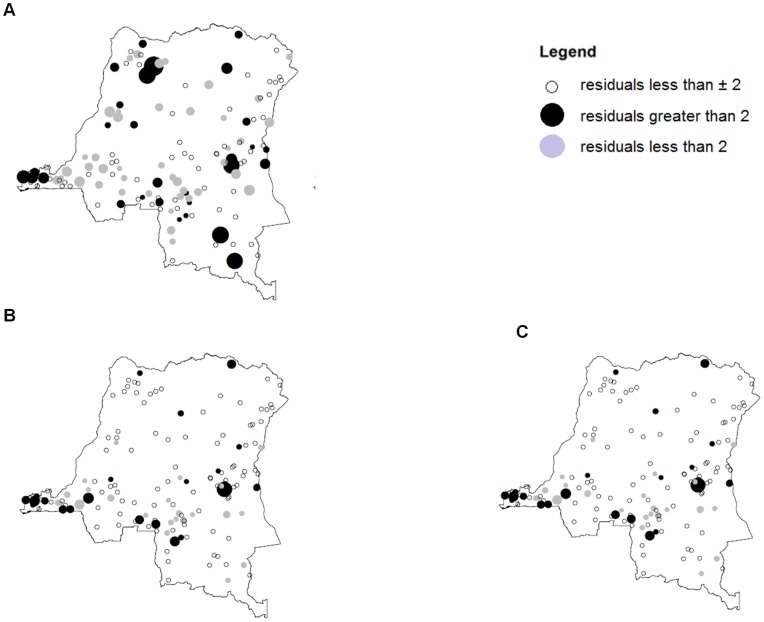
Residual Maps. Maps of the residual obtained by fitting a logistic regression model to A) any bed net coverage, B) ITN coverage and C) ITN density. Black circles indicate residuals greater than 2, and grey circles indicate residuals smaller than −2. The size of the black and grey circles are proportional to the absolute value of the residuals.

Figure 4 presents maps of the standardised deviance residuals obtained from the regression models fitted to the bed net coverage, ITN coverage and ITN density measures. The three maps highlight the large positive residuals observed around the Bas-Congo province near Kinshasa, thus indicating that the models were generally under-predicting bed net ownership in this area. The pattern and size of the residuals for the remainder of the country appeared to be more random. The global Moran's I tests conducted on the residuals from each of the three models were significant with p-values <0.02, with rural clusters in the Bas-Congo province being identified as a spatial cluster of positive residuals. This suggested that the selected models were not able to explain the higher levels of bed net ownership in areas around the capital city, Kinshasa. Therefore, an additional term was included in the model in order to account for these observations, namely an indicator variable that was equal to 1 when the cluster was within the Bas-Congo province, and zero otherwise. The inclusion of this term significantly improved the fit of all three models, and further resulted in the ‘distance to railway’ term in all three models being removed. Moran's I tests performed on the residuals from each of the updated models were no longer significant, indicating that no spatial autocorrelation remained in the residuals.

The resulting adjusted odds-ratio (OR) of each factor and their associated 95% confidence intervals (95% CIs) for the coverage models are shown in Table 3, whereas Table 4 presents the relative risk (RR), plus associated 95% confidence intervals for the density model As the number of ITNs per cluster is very small in comparison to the surveyed population size, RR can be considered to be a good estimate of OR, hence the results in Tables 3 and 4 are comparable [37]. These results show that the ‘Bas-Congo effect’ had a large impact on bed net ownership, particularly for ITNs. For example, the ITN coverage in the Bas-Congo is 5.3 times larger (95% CI 3.67–7.70) than those outside of the Bas-Congo after controlling for other factors. The OR/RR values associated with the distance to health facilities, major cities and transport links are all less than one, indicating that as the distances from these features increased there was a reduction in bed net ownership. The cumulative effect of distance from the two main cities is similar for all three bed net ownership measures. The remaining OR/RR values obtained for the variables included in the two ITN-related models were also very similar, indicating that the conclusions drawn are not dependent on our method of summarising ITN ownership.

**Table 3:** Logistic regression model results for bed net coverage.

Variable	OR	95% CI		p-value
**Any bed net coverage**				
** I(Bas-Congo province)**	2.59	2.01	3.35	<0.0001
** log(Distance to Kinshasa/** ** Lubumbashi (km))**	0.77	0.67	0.88	0.0002
** log(Distance to national** ** road (km))**	0.92	0.88	0.96	0.0005
** log(Distance to main** ** waterways (km))**	0.75	0.69	0.8	<0.0001
** ITN coverage**				
** I(Bas-Congo province)**	5.29	3.67	7.7	<0.0001
** log(Population density)**	1.11	0.99	1.26	0.0804
** log(Distance to Kinshasa/** ** Lubumbashi (km))**	0.72	0.59	0.88	0.0011
** log(Distance to health** ** facility (mins))**	0.91	0.83	0.99	0.0238

**Table 4:** Poisson regression model results for ITN density.

Variable	RR	95% CI		p-value
**ITN density**				
** I(Bas-Congo province)**	4.67	3.42	6.43	<0.0001
** log(Population density)**	1.10	0.99	1.22	0.0877
** log(Distance to Kinshasa/** ** Lubumbashi (km))**	0.78	0.66	0.93	0.0041
** log(Distance to health** ** facility (mins))**	0.91	0.84	0.97	0.0076

#### Interactions

An exploratory analysis of the interaction between population density and distance to Kinshasa/Lubumbashi, in which clusters were categorised as either within or greater than 400 km of the two cities, and with population density greater or less than the median of 23 people per km^2^, was conducted. The results are tabulated in Table S2, and indicate that when the cluster is far from Kinshasa/Lubumbashi, the bed net ownership is low irrespective of population density, whereas when clusters are close to the cities, ownership is greater in the more densely populated areas in comparison to the sparsely populated areas. Note that there are only 37 clusters within 400 km of Kinshasa/Lubumbashi, 13 of which have low density, therefore there is a large amount of uncertainty attached to the summaries in this category. Therefore, a more formal test for interactions was undertaken with a ‘population density/distance’ term in the models without the ‘Bas-Congo effect’. This term was found to be significant in the ITN ownership models only. However, on comparing this interaction model with the ‘Bas-Congo effect’ model, the latter model was determined to better represent the data as determined by a smaller Akaike Information Criterion (AIC) [38].

## Discussion

This study highlights the distribution and disparities in bed net ownership across DRC, one of the poorest and most populated countries in sub-Saharan Africa, with one of the greatest burdens of LF in the world [1]. The results suggest that for the national LF Programme to fully take advantage of bed nets as an alternative or additional strategy in this vast country, there needs to be continued efforts to understand the limiting factors, and better integration with malaria programmes to improve net distribution. The WHO statement recommending IVM control in countries that are co-endemic with both LF and malaria should assist in redressing this imbalance of the countries with the heaviest burden receiving the least amount of treatment [10], and new strategies for interrupting LF in loiasis endemic countries will also help DRC move forward. In addition, WHO recommends that IVM should be considered to be a two-way strategy i.e. the infrastructure developed for MDA for LF and other diseases such as onchocerciasis should be used to expand the distribution of ITNs in hard-to-reach areas [10], [39]. For example, by 2009 the African Programme for Onchocerciasis Control (APOC) had treated 85.9% of targeted communities within DRC with the community-directed treatment with ivermectin (CDTi) strategy [40]. A crucial element of this strategy is the recruitment of community-directed distributors, and if this system were to be further utilised by vector control programmes, then geographical coverage of bed nets could be vastly improved.

Significant differences were found in bed net ownership between urban and rural areas. These differences may be attributed to their distinctive demographic and infrastructure characteristics, and potentially amplified by the conflict that left the country severely weakened both politically and structurally, with poor access to basic services such as health care and transport. Individuals living in rural areas were found to travel, on average, four times (or 40 mins) longer to reach the nearest health facility, nine times (or 130 km) further to the nearest railway, and more than 25 times further to reach both the nearest main road (or 83 kms) or the capital city of Kinshasa (or 358 km) than those living in urban areas. It is therefore not surprising that urban areas had higher levels of bed net ownership, especially as many were within close proximity to one of the three main economic cities. In particular, Kinshasa situated on the Congo River, and Lubumbashi a heavily industrialised city within the copper-mining area of DRC (close to Zambia), appear to be important urban hubs, and their strategic geographical locations and interconnected main national road and rail networks running through the populated southern half of the country are likely to have influenced the higher coverage in urban areas.

Key to the success of vector control programmes will be the delivery of bed nets to the entire at-risk population, including those in remote hard-to-reach areas [41]. Accessing these areas will greatly be assisted by an appropriate and efficient transport system, and there are many challenges for DRC given the general lack of development, and the deterioration in infrastructure from years of civil unrest. However, as the stability improves in DRC, there are reports of greater investment and plans to extend the existing transport structures across the country e.g. the World Bank Multi-modal Transport project [42]. Such investments are encouraging and should be utilised by national control programmes to access a larger proportion of the at-risk populations. However, it will be important to take the unique geographical features of DRC into consideration comprising of mountainous regions in the far west and along the eastern border, and the majority of the country being occupied by the extensive Congo River Basin. In this area, many *Anopheles* mosquito species have been found [28] and the risk of LF is thought to be greatest [4], [43].

Accessing the remote rural areas in the Congo River Basin through navigable waterways may be logistically easier and more appropriate than road and rail networks. We found a negative relationship between any bed net coverage and distance to the main navigable waterways, which suggests that the use of the extensive river system may be particularly useful to distribute such vector control tools. Although we did not find the same relationship with ITNs, we attribute this to ITN ownership being generally low throughout the entire country, including the entirety of the Congo River Basin. Given the large geographical size of the Basin, the use of air transport to distribute bed nets to high risk areas should be considered. Whilst no relationship was observed between the distance to the nearest airport and bed net ownership, possibly due to due to airports being relatively densely situated throughout the whole country, this mode of transport may be suitable for selected LF and *Loa loa* co-endemic areas where the risk of SAEs is high and the need for alternative strategies and methods of delivery is a priority [11]. Clearly, innovative ways to reach remote areas are urgently needed. The use and expansion of the CDTi strategy and distribution networks used by APOC may be the crucial link to improving the coverage of bed nets and other interventions for neglected tropical diseases and malaria across DRC.

The results of the multivariate analysis showed that differences in bed net ownership, in particular ITNs, within rural areas can partly be explained by differences in population density, the distance to health facilities and distances to the main cities of Kinshasa and Lubumbashi in the populous southern region of DRC. Further, after accounting for the effects of these variables, bed net ownership in rural areas of the Bas-Congo province was much higher than the rest of the rural areas in the country causing a ‘Bas-Congo effect’ in the distribution pattern. This may be due to the Bas-Congo being one of the smallest yet more densely populated provinces of DRC that is close to the capital, Kinshasa, and has more accessible transport links including access via National Road 1, which connects Kinshasa to the main sea ports of Boma and Matadi. The significantly higher distribution of bed nets and ITNs in rural areas of the Bas-Congo province may be viewed as encouraging indicator of efforts made to distribute bed nets outside of urban areas. Moreover, these and subsequent ITN distributions [44] are likely to have significantly impacted LF in this province, which has historically shown to be highly endemic for LF transmitted by *Anopheles funestus* [4].

Differences between the variables included in the logistic regression models for any bed net coverage and logistic regression and Poisson regression ITN models suggest that ITN ownership was not uniformly proportional to bed net ownership across the country. This is perhaps an indication that ITN distribution campaigns up to the time of the DHS survey had been focused on those areas that were easy-to-reach, whereas bed nets in general at this time were more ubiquitous across the country. Since 2007 there has been a significant scale up of malaria prevention in DRC and a report in 2010 [45] indicated that 51% of households owned at least one ITN (58% urban 48% rural). This increase in ITNs is promising, however, the provincial survey data available indicated that disparities still occur across the country, which have been attributed to the poor quality of the transport system and coordination problems resulting from a weak communication network [28]. Continued efforts to understand and resolve these disparities are critically important for both the national malaria and LF programme.

In undertaking this analysis the assumption has been made that the DHS data provide an accurate representation of the urban and rural populations of DRC. Whilst robust sampling method have been used in undertaking the surveys, a major limitation to the survey design was that the sampling frame used was based primarily on 1984 census data. Due to the conflicts that ravaged the country during the 1990's and early 2000's, the demographic landscape is likely to have changed vastly since this period, with the National Institute of Statistics in DRC estimating that the population will have more than doubled from 30.7 million in 1984 to 65.8 million in 2007, with the urban population increasing from approximately 30% of the population to 40–45% of the population [27]. However, as the country's stability increases, and the infrastructure improves, data such as that obtained by the DHS will become more reliable, and the imminent Population and Housing census will enable the true changes in DRC's demography to be quantified.

Measuring the distribution and impact of ITNs is becoming increasingly important for national control programmes, international stakeholders and funding organisations, suggesting that a standardised ITN tracking system or centralised database relating to where and when ITNs are being distributed and the population covered would be valuable [28]. Here, we examined two measures of ITN ownership. Firstly, the proportion of households with at least one ITN was considered. This is a commonly used measure amongst policy makers and within the literature, but does not take household size into account, and as a result may produce optimistic summaries. Secondly, the ITN density per cluster was examined. This may be a more accurate representation of bed net ownership and the related protection provided to the population. Studies have shown a positive relationship between bed net usage and bed net density at the household level [46]–[48], with bed net usage being between 2 and 5 times higher when net density was greater than 0.5 compared to when it less than 0.5 [48]. The latter density measure is currently used by international malaria programmes [4], and could equally be used by GPELF and international LF programmes, with the term ‘universal coverage’ for ITNs integrated into current LF protocols for MDA coverage where appropriate.

Whilst the influence of demographic features and transport networks has been used to explore bed net ownership in DRC on a small spatial scale [19], this analysis demonstrates that these relationships persist at a much larger spatial scale across a country with a very diverse geography. Other studies have considered the influence of a number of these community-level variables (distance to town, health facility, road, river), plus household-level bed net ownership on malaria prevalence in DRC [21], [22] however these results are not applicable to other diseases. The advantage of our study focusing on the distribution of vector-control tools rather than a particular disease is that these results will be of interest to multiple disease programmes needing to scale-up and distribute their interventions across this unique country. The national LF programme in DRC will gain from these insights, and whilst the analysis explains some of the variability in bed net ownership, it will be critical to consider the numerous other non-spatial factors such as wealth, education, knowledge of the disease and additional social factors [18], [49], [50] that may affect the impact of bed nets in accelerating the elimination of *Anopheles*-transmitted lymphatic filariasis in hard-to-reach communities.

## Supporting Information

Table S1 **DHS and GRUMP urban/rural classification summaries.** Summaries of bed net ownership and demographic and transport network variables by DHS and GRUMP urban/rural classification.
(PDF)

Table S2 **Rural bed net ownership by population density and distance from Lubumbashi/Kinshasa.** Bed net ownership summaries by population density category (greater or less than 23 per km^2^) and distance from Lubumbashi/Kinshasa category (greater or less than 400 km) for rural clusters.
(PDF)
