# Supplementary material for: Geographical Factors Affecting Bed Net Ownership, a Tool for the Elimination of Anopheles-Transmitted Lymphatic Filariasis in Hard-to-Reach Communities
Source: PLoS One. 2013 Jan 7;8(1):e53755. doi: 10.1371/journal.pone.0053755 (PMC3538722; doi:10.1371/journal.pone.0053755)
Supplement: Table S1 — DHS and GRUMP urban/rural classification summaries. Summaries of bed net ownership and demographic and transport network variables by DHS and GRUMP urban/rural classification. (PDF) [file pone.0053755.s001.pdf]

Table S1: DHS and GRUMP urban/rural classification summaries. Summaries of bed net ownership and demographic and transport network variables by DHS and GRUMP urban/rural classification.

	DHS category						GRUMP category					
	Urban (n=115)			Rural (n=145)			Urban (n=80)			Rural (n=180)		
Attribute	Median	IQR		Median	IQR		Median	IQR		Median	IQR	
Bed net summaries												
Any bed net coverage	43	17	53	17	7	40	43	19	53	17	7	43
ITN coverage	10	3	20	3	0	10	10	7	20	3	0	11
ITN density (per 100 pop.)	2	1	4	1	0	2	2	1	4	1	0	2
Demographic features												
Pop. density	210	23	6366	23	10	41	1112	79	7953	23	10	43
Distance to health facility	17	10	21	55	15	120	15	10	20	31	15	95
Distance to nearest major city				491	307	626	17	7	580	480	281	626
Distance to Kinshasa	691	15	1210	975	538	1306	526	10	1190	956	481	1305
Distance to Kinshasa/Kisangani	393	14	754	525	374	685	334	8	685	520	323	739
Distance to Kinshasa/Lubumbashi	365	10	806	745	399	959	190	8	756	732	334	958
Transport features												
Distance to national Road	5	2	145	88	40	231	3	1	87	86	32	227
Distance to railway	23	8	165	147	57	246	16	7	88	138	50	242
Distance to main waterways	150	8	363	252	128	375	13	7	361	256	128	386
Distance to airport	9	5	80	114	76	155	7	4	11	107	62	148
