# Supplementary material for: Geographical Factors Affecting Bed Net Ownership, a Tool for the Elimination of Anopheles-Transmitted Lymphatic Filariasis in Hard-to-Reach Communities
Source: PLoS One. 2013 Jan 7;8(1):e53755. doi: 10.1371/journal.pone.0053755 (PMC3538722; doi:10.1371/journal.pone.0053755)
Supplement: Table S2 — Rural bed net ownership by population density and distance from Lubumbashi/Kinshasa. Bed net ownership summaries by population density category (greater or less than 23 per km2) and distance from Lubumbashi/Kinshasa category (greater or less than 400 km) for rural clusters. (PDF) [file pone.0053755.s002.pdf]

Table S2: Rural bed net ownership by population density and distance from Lubumbashi/Kinshasa. Bed net ownership summaries by population density category (greater or less than 23 per km^2) and distance from Lubumbashi/Kinshasa category (greater or less than 400km) for rural clusters.

Interaction	Median	95% CI	
**Any bed net coverage**			
Cities >400km, pop. density >23	13	10	30
Cities >400km, pop. density <23	13	3	39
Cities <400km, pop. density >23	37	7	53
Cities <400km, pop. density <23	15	10	53
**ITN coverage**			
Cities >400km, pop. density>23	3	3	7
Cities >400km, pop. density<23	0	0	3
Cities <400km, pop. density>23	16	3	36
Cities <400km, pop. density<23	10	5	10
**ITN density (per 100 pop.)**			
Cities >400km, pop. density >23	2	1	1
Cities >400km, pop. density <23	0	0	1
Cities <400km, pop. density >23	4	1	1
Cities <400km, pop. density <23	1	1	1
